# Non-tuberculous mycobacterial lung disease: a brief review focusing on radiological findings

**DOI:** 10.1590/0037-8682-0241-2020

**Published:** 2020-09-11

**Authors:** Laura Raniere Borges dos Anjos, Poliana Lopes Parreira, Pedro Paulo Teixeira Silva Torres, André Kipnis, Ana Paula Junqueira-Kipnis, Marcelo Fouad Rabahi

**Affiliations:** 1 Universidade Federal de Goiás, Instituto de Patologia Tropical e Saúde Pública, Departamento de Biociências e Tecnologia, Goiânia, GO, Brasil.; 2 Universidade Federal de Goiás, Faculdade de Medicina, Departamento de Clínica Médica, Goiânia, GO, Brasil.

**Keywords:** Non-tuberculous mycobacteria, Lung disease, Computed tomography, Radiological findings

## Abstract

The incidence and prevalence of lung disease caused by non-tuberculous mycobacteria (NTM-LD) has increased worldwide and its diagnosis represents a complex challenge. This article aims to review the tomographic findings of NTM-LD in order to facilitate their definitive diagnosis. The search for publications on the subject was performed in PMC and Scielo using the keywords ‘non-tuberculous mycobacteria’, ‘lung disease and computed tomography (CT)’ and ‘radiological findings’. The radiological findings described by 18 articles on mycobacteriosis were reviewed. In addition, CT images of patients diagnosed with NTM-LD were considered to represent radiological findings. Eighteen publications were used whose main findings were pulmonary cavitation (88.9%), bronchiectasis (77.8%), and pulmonary nodules (55.6%). Despite the overlaps in imaging-related analysis of myocobacterioses with other pulmonary infections, such as tuberculosis, the predominant involvement of the middle lobe and lingula should raise suspicion for NTM-LD.

## INTRODUCTION

Mycobacteria are a unique genus, called *Mycobacterium*, which are members of the family *Mycobacteriaceae* and the order *Actinomycetales* ^1^ ^,^ ^2^ *.* It is speculated that mycobacteria emerged 150 million years ago and so far over 170 different species and subspecies have been identified. These species can be organized into three groups: *Mycobacterium tuberculosis* complex (MTBC), *M. leprae,* and non-tuberculous mycobacteria (NTMs)^3^.

NTMs, previously identified as atypical mycobacteria, are defined as a heterogeneous group of species with distinct genotypic and phenotypic characteristics than MTBC or *M. leprae* ^4^ ^,^ ^5^. NTMs are geographically distributed heterogeneously and can be found in animal species, vegetation, biofilms and especially in water and soil^4^ ^,^ ^6^ ^,^ ^7^.

The human pathogenicity of NTMs has been of interest to the scientific community, but this has not been the case always^8^. The first description of pathogenic NTMs occurred in 1950 when around 2% of patients initially diagnosed with tuberculosis (TB) did not respond to traditional treatments and were subsequently diagnosed with mycobacterial infections that did not cause tuberculosis^8^ ^,^ ^9^. Since then, other studies are being conducted to improve the understanding of the pathophysiological mechanisms involved in NTM-related diseases^10^. 

Non-tuberculous lung disease (NTM-LD) is the most common infection caused by NTMs, especially in immunosuppressed individuals. NTM-LD is not reportable to public health authorities and therefore it is difficult to estimate its epidemiological characteristics. Nevertheless, it is noted that the incidence and prevalence of NTM-LD has been increasing considerably in several regions of Brazil^4^. It is also suspected that other regions are experiencing a silent epidemic of this disease^4^ ^,^ ^11^.

Additionally, the diagnosis of NTM-LD requires compatible clinical and radiological findings, along with two or more positive sputum samples for the same NTM species or one positive bronchial wash/lavage or compatible histopathological findings with at least one positive culture^12^. Fungal infections or other mycobacterial infections might present similar clinical manifestations and radiological findings and so they pose an additional challenge for the correct microbiological identification and diagnosis^12^ ^-^ ^14^. Hence, NTM-LD represents a complex challenge for patients, health care, and health authorities around the world^12^.

This article reviewed the radiological findings described in other articles on MNT-LD to identify the major pulmonary changes presented by these patients. The results obtained could contribute in differentiating between MNT-LD and TB and can facilitate the diagnosis of MNT-LD.

## METHODOLOGY

For this review, the search and selection of publications was performed on the following platforms: *Pubmed Central* (PMC) and *Scientific Eletronic Library Online* (SciELO). The search terms used were ‘non-tuberculous mycobacteria’, ‘lung disease and computed tomography’, and ‘radiological findings’.

The inclusion criteria for articles were those published in Portuguese, English or Spanish from 1999 to 2019. The reference lists of all retrieved articles were checked to identify other eligible publications. Comments, gray literature and other publications that did not meet the inclusion criteria were excluded.

**FIGURE 1:**
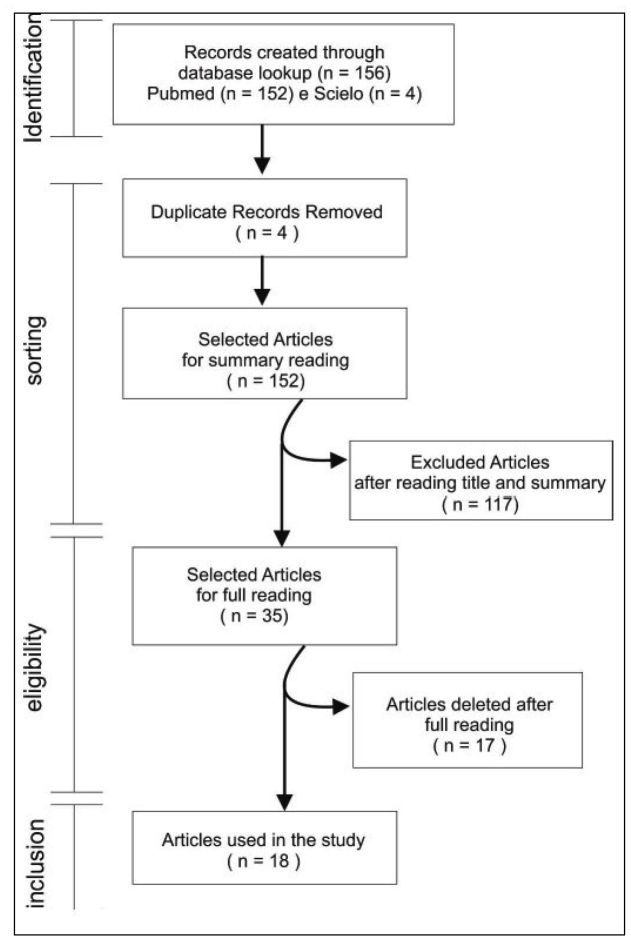
A flow diagram describing the stages of article identification, screening, eligibility and inclusion which are considered in this brief review.

**FIGURE 2:**
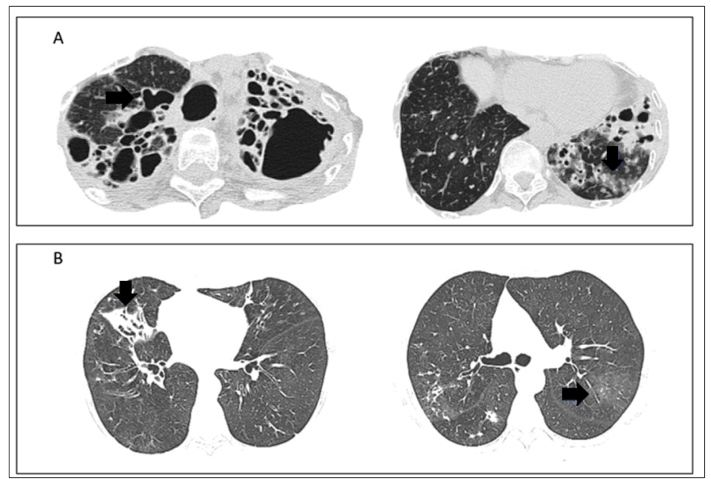
(A) CT scan of an immunocompromised patient with *M. asiaticum*; extensive varicose bronchiectasis is observed in a severe form of the disease associated with bronchial parietal thickening (black arrow). Centrilobular opacities with attenuation in “ground-glass” are also visible (black arrow). (B) CT scan of patients with *M avium-intracelulare*; consolidation of the middle lobe. The associated bronchiectasis predominance of the middle lobe and lingual are noted (black arrow). Centrilobular opacities with attenuation in “ground-glass” is also evident (black arrow).

**FIGURE 3:**
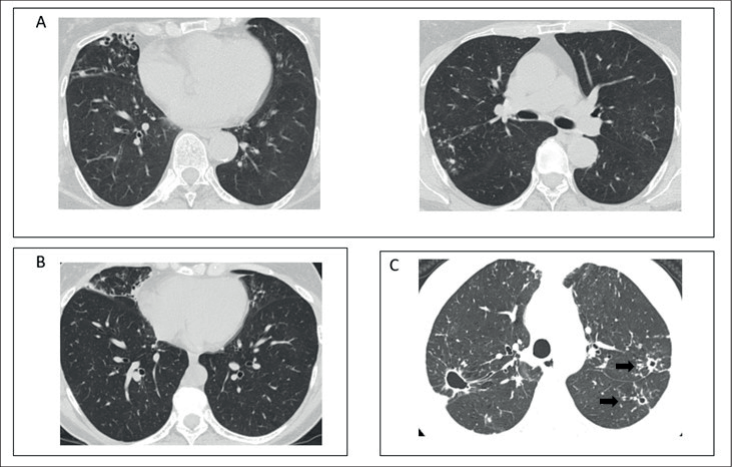
(A) CT scan of a patient with *M. fortuitum*; bronchiectasis in the middle lobe is associated with micronodules in the right upper lobe. (B) CT scan of a patient with *M. intracelulare;* bronchiectasis and micronodules is visible in the middle lobe and lingula. (C) Cavitation with thick walls in the upper lobes and centrilobular micronodules and some with “budding tree” pattern in the left upper lobe (black arrows).

The flow diagram of this review is detailed in Figure 1. In total, 18 articles were considered and all the radiological findings are summarized in Table 1. To represent tomographic findings, computed tomography (CT) images (Figure 2 and Figure 3) obtained from archives of medical assistants in the region of Goiânia, Goiás, Brazil, were used. It is important to highlight that these images were set as anonymous by the professionals in charge before being made available for this review.

**TABLE 1:** Radiological findings of CT scans from patients with NTM-DP.

Author	Region/Country	Number of patients	Methodology^2^	Radiological findings^3^
(Year of publication)		with NTM infection^1^		
Fujita et al. (1999)^36^	Kagawa, Japan	5	CT	bronchiectasis; cavitation; centrilobular nodules; consolidation; bronchiolitis;
Koh et al. (2002)^46^	Seoul, Korea	(-)	CT	MAC: upper lobe cavitation; nodules; bronchiectasis; progressive fibrosis with volume loss. *M. kansasii:* cavitation with a preponderance of upper lobe; parenchymal infiltration.
Shitrit et al. (2008)^49^	Israel	*M. simiae* (*n*= 102)	CT	*M. kansasii:* cavitation and a predilection for upper lobe; infiltration. *M. simiae:* infiltration, cavitation; pleural effusion; lymphadenopathy
		*M. kansasii* (*n*= 62)		
Park et al. (2010)^37^	Seoul, Korea	41	CT	Nodules; consolidation; cavitation; bronchiectasis; pleural effusion; lymphadenopathy
Matveychuk et al. (2012^58^	Tel Aviv, Israel	98	CT	*M. kansasii:* more cavitations, unilateral disease, right upper lobe disease more common**;** pleural effusion; lymphadenopathy; Other NTM: infiltration and cavitation; lower and middle lobe predominance; pleural effusion; lymphadenopathy
Baghaei et al. (2012)^38^	Tehran, Iran	26	CT	*M. simiae*: Nodular lesion; cavitation; bronchiectasis
Kobashi et al. (2013)^52^	Kawasaki, Japan	220	CT	*M. avium, M. intracellulare* and *M. kansasii*: bronchiectatis; cavitation; pneumothorax;
Gommans et al. (2015)^50^	Maastricht/The Netherlands	124	CT	MAC: cavitation; bronchiectasis; consolidation; *M. kansasii:* cavitations, nodules, bronchiectasis consolidation; *M. malmoense:* cavitations, consolidation; MCR: cavitation, nodules; Non-MNT findings: atelectasis; pleural effusion; tumors
Yoon et al. (2016)^59^	Seoul, Korea	5	CT	MAC: apical fibrocavitary disease; nodular infiltrates frequently involving the right middle lobe and the lingula; pleural hydropneumothorax; consolidation; tree-in-bud pattern; bronchiectasis; pleural effusion; pleural thickening
Hwang et al. (2017)^51^	Seoul, Korea	488	CT	bronchiectasis and small centrilobular nodules predominantly in the right middle lobe or lingula; apical fibrocavitary lesions
Ueyama et al. (2016)^41^	Tokyo, Japan	69	CT	Nodule; bronchiectasis; consolidation; subpleural thickening; interlobular septal thickening; cavitation; pleural effusion
Kwak et al. (2016)^48^	Seoul, Korea	66	CT	Atelectasis; ground-glass opacity; bronchiectasis; cavitation; nodular and micronodular lesions; tree-in-bud pattern; consolidation; pleural effusion
Monteiro et al. (2018)^43^	Pará, Brazil	43	CT	Cavitation; bronchiectasis; fibrocavitary lesions
Cowman et al. (2018)^47^	London, United Kingdom	85	CT	*M. abscessus*, MAC, *M. kansasii*, *M xenopi* and other species of NTMs: cavitation; nodules; bronchiectasis; tree-in-bud changes
Hirama et al (2019)^42^	Toronto, Canada	94	CT and X-ray	cavitation; bronchiectasis; centrilobular nodules/tree-in-bud; random nodules; consolidation/ground glass opacity; pleural effusion; pleural thickening; mediastinal lymphadenopathy
Cowman (2018)^40^	London, United Kingdom	(-)	CT	tree-in-bud; consolidation; atelectasis; fibrotic changes; volume loss and pleural thickening
Bakuła et al. (2018)^60^	Warsaw, Poland	105	CT and X-ray	Infiltration; interstitial pattern/fibrosis; cavitation; nodules; bronchiectasis; massive fibrotic lesions; pleural effusion; mediastinal lymphadenopathy
De Marca et al. (2019)^53^	Rio de Janeiro, Brazil	48	CT	*M. kansasii,* MAC, *M. fortuitum, M. gordonae, M. abscessus*: architectural distortion; reticular opacities; bronchiectasis; cavitation; centrilobular nodules; atelectasis; small and large consolidations.

1 - (-) is a review article with no patients; 2 - CT: computed tomography; 3 - MAC: *Mycobacterium avium* complex.

## EPIDEMIOLOGY

Considering the epidemiological data of NTM-LD available in the literature, it is observed that the disease prevalence varies between regions. In general, the epidemiology of this disease accompanies the distribution characteristic of NTM species^4^.

Based on the surveyed literature, the most frequent species are *M. kansasii* and the *M. avium* complex (MAC), which includes the subspecies *M. avium*, *M. intracellulare,* and *M. chimaera*. These pathogens are very common in Europe (80% of cases), North America, South Korea and are often associated with LD cases that are similar to TB^6^ ^,^ ^15^ ^-^ ^17^ *.*

*M. xenopi* is also associated with LD and is found in Europe and in certain regions of Canada. In the Middle East, the most common clinical isolate is *M. simiae*, which can cause infection in various organs, especially the lung^6^ ^,^ ^15^ ^,^ ^18^. 

Recently, other NTMs have emerged as human pathogens, including *M. fortuitum*, *M. chelonae* and the *M. abscessus* complex (MABSC)^6^ ^,^ ^19^. Genome sequencing technology has enabled the taxonomic organization of the MABSC into three subspecies: *M. abscessus* subsp. *bolletii, M. abscessus* subsp. *abscessus* and *M. abscessus* subsp. *massiliense*, and these are common in Asia, Oceania and South Korea^11^ ^,^ ^15^ ^,^ ^20^ ^,^ ^21^. These emerging NTMs can cause skin, soft tissue, and central nervous system infections and can also lead to LD^19^ ^,^ ^20^ ^,^ ^22^ ^,^ ^23^. LD caused by MABSC mainly affects patients with predisposed diseases such as cystic fibrosis^20^ ^,^ ^24^. 

## TRANSMISSION AND CLINICAL MANIFESTATIONS

NTM-LD guidelines include the various forms of transmission of the etiological agent. For a long period of time, it was accepted that the transmission of NTMs occurred through the environment. It was later reported that some species were related to medical devices used in surgical procedures or esthetic treatments^25^. Recently, direct transmission of NTM species among cystic fibrosis patients has also been documented^20^. 

Once transmitted, NTMs can settle in any organ and can develop an asymptomatic infection or can lead to a disseminated disease that can be fatal to humans^25^ ^,^ ^26^. The lung is the target organ of NTM-LD and its involvement implies different clinical manifestations that negatively impact patients' quality of life^27^. It is described in the literature that NTM-LD might occur in three prototypic forms: fibrocavitary disease, nodular bronchiectasia, and hypersensitivity pneumonitis^28^, and the radiological findings of these three forms were described in various articles as cited in Table 1.

Signs and symptoms of fibrocavitary disease have traditionally included nonspecific symptoms such as purulent sputum, dyspnea, hemoptysis, chest pain, fever, asthenia, weight loss, shortness of breath, and night sweats^10^ ^,^ ^23^ ^,^ ^29^. It is common for the patient with this manifestation to also develop underlying diseases such as TB, chronic obstructive pulmonary disease (COPD), or pneumoconiosis^10^ ^,^ ^16^ ^,^ ^29^ ^,^ ^30^. Fibrocavitary disease is more common in males over 50 years of age and among Caucasian population and smokers^28^. Advanced cases might result in respiratory failure within 1 to 2 years after the onset of the disease. This predisposing condition is responsible for the highest mortality rate caused by NTM-LD^29^ ^-^ ^31^. 

Nodular bronchiectasis has a much slower progression rate and typically has no underlying LD^32^. Furthermore, hypersensitivity pneumonitis is considered an inflammatory reaction caused by the inhalation of large quantities of aerosols containing NTMs^29^ ^,^ ^33^. The signs and symptoms of nodular bronchietasia and hypersensitivity pneumonitis are similar to fibrocavitary disease and in all cases, the diagnosis is made from evidences found via chest radiography or CT^28^ ^,^ ^29^.

## RADIOLOGICAL DIAGNOSIS

Current diagnostic criteria of NTM-LD were established in 2007 by the American Thoracic Society (ATS) and the Infectious Diseases Society of America (IDSA). Since then, diagnosis is based on clinical evidence, microbiological identification, and radiological findings^4^ ^,^ ^34^. Various studies describing the tomographic appearance of NTM-LD have been performed over time and these are listed in Table 1.

This review noted that 77.8% of the articles on NTM-LD published between 1999 and 2019 described bronchiectasis among their CT findings (Table 2). Bronchiectasis is characterized by permanent and abnormal dilation of the bronchi triggered after persistent bacterial airway infection^35^. Figure 2A and Figure 2B show representative images of CT scans showing bronchiectasis in an anonymous patient who was diagnosed with NTM-LD according to the ATS and IDSA criteria.

A study by Fujita et al. (1999) suggests that discrete nodule bronchiectasis is an important radiological finding of MAC infection and colonization^36^. Another study conducted with the Korean population revealed that bronchiectasis was associated with lung colonization by *M. kansasii* in LD patients^37^. Baghaei et al. (2012) also associated bronchiectasis with LD caused by *M. simiae* among the Iranian population^38^. 

Another study by Bonnet et al. (2017) showed that approximately two-thirds of patients with NTM-LD also had fibrocavitary disease with bronchiectasis (60%) and cavitation (40%)^39^. Bronchiectasis is also among the most predominantly identified radiological observations in CT scans of patients with NTM infection as described in a study conducted by Cowman and Loebinger (2018)^40^. 

Ueyama et al. (2016) also reported that bronchiectasis was observed via CT images among 96% of adult patients with NTM-LD evaluated in their study^41^. In the study by Hirama, Brode and Marras (2018), it was identified that 42.6% of the patients with LD by *M. xenopi* also presented nodular bronchiectasis based on CT scans^42^. Interestingly, in this retrospective study, patients with nodular bronchiectasis were predominantly female. These patients had characteristics which were suggestive of milder disease and were treated less frequently. In addition, these patients received fewer anti-mycobacterial drugs during treatment. Taken together, these results were not sufficient to determine a specific radiological pattern for LD by *M. xenopi* ^42^.

In Brazil, a study on clinical aspects of patients with LD by MABSC identified the presence of nodular bronchiectasis in individuals diagnosed with NTM infection but were not undergoing any treatment. However, these results are not sufficient to determine a radiological standard for NTM-LD findings^43^. 

It should be noted that some of the studies cited here were performed in patients with cystic fibrosis who had a predisposition to changes such as bronchiectasis^44^. Hence, it is not possible to state whether the onset of bronchiectasis favors lung colonization by NTMs or vice versa^45^ ^,^ ^46^.

This characterization of bronchial alterations as an important radiological finding of NTM-LD is relevant for the diagnosis and also for the patient recovery. A study by Cowman et al. (2018) suggested that bronchiectasis interfered with the duration of the disease and was associated with severe lung disease prognosis^47^. 

 Another obstacle for the radiological diagnosis of NTM-LD was highlighted by Bonnet et al. (2017) where the authors showed that CT scans from TB patients also feature post-inflammatory bronchiectasis^39^. According to Kwak et al. (2016), NTM-LD and pulmonary TB bronchiectasis are difficult to distinguish and, therefore, it is necessary to observe other radiological characteristics^48^, such as, cavities and distribution of pulmonary changes in CT images. Compared with pulmonary TB, NTM-LD tends to form cavities less frequently^56^ and involves more of the middle and/or lower lung regions and bilateral lungs more frequently^57^. 

This review found that 55.6% and 88.9% of the articles indicate presence of pulmonary nodules and presence of cavitation on CT images of patients with NTM-LD, respectively (Table 2). Figure 3C represents CT images of patients with NTM-LD who presented cavitation and pulmonary nodules based on radiological findings.

Pulmonary nodules were also identified in 98% of adult patients with NTM-LD evaluated in the study by Ueyama et al. (2016). Interestingly, in that study, other findings were also reported, including cavitation ( in 77% of patients)^41^. 

Studies by Shitrit et al. (2008) and Matveychuck et al. (2012) conducted among Israeli population revealed numerous upper lobe cavitations in patients with *M. kansasii* infection^49^ ^,^ ^58^. In a subsequent study, the prevalence of thin-walled cavities in upper lobes of patients with infections caused by NTMs was also observed. In these cases, some pleural abnormalities around the cavities were observed^16^. 

In the study by Shitrit et al. (2008), pleural abnormalities and lymphadenopathy were also identified. However, in this study, these abnormalities were only associated with the presence of pulmonary infiltrates located in the middle and upper lobes among patients with infections caused by *M. simiae* ^49^. On the other hand, it has been reported that MTBC is also capable of promoting cavitation formation. Thus, it was concluded that cavitations are nonspecific alterations of NTM-LD and might reflect infections caused by several pathogens commonly found in diseases that compromise airway functions^4^ ^,^ ^24^. 

This review identified other radiological findings such as pleural effusion and mediastinal lymphadenopathy, which were observed in a study by Bakula et al. (2018). Other findings of this study were consolidation (50%), ground-glass opacities (33.3%), other pleural diseases (22.2%), pleural lymphadenopathy (27.8%), atelectasis (22.2%), “budding tree” pattern (22.2%), fibrotic alteration / volume loss / pulmonary architecture distortion (38.9%), hydropneumotorax / pneumothorax (11.1%), and bronchiolitis (5.6%). All the findings are summarized in Table 2.

**TABLE 2:** Frequency of studies that report radiological findings among NMT infections as described in the articles included in this review.

Radiological finding	Number of studies that identified^*^		Number of studies that did not identify		Total studies
	n	%	n	%	n
Pulmonary cavitation	16	88.9	2	11.1	18
Bronchiectasis	14	77.8	4	22.2	18
Pulmonary nodules	10	55.6	8	44.4	18
Consolidation	9	50.0	9	50.0	18
Pleural effusion	9	50.0	9	50.0	18
Fibrotic alteration/loss of volume/architectural distortion	7	38.9	11	61.1	18
Ground-Glass Opacities	6	33.3	12	66.7	18
Lymphadenopathy	5	27.8	13	72.2	18
Others Pleural Diseases	4	22.2	14	77.8	18
Atelectasis	4	22.2	14	77.8	18
“Budding tree” pattern	4	22.2	14	77.8	18
Hydropneumothorax /pneumothorax	2	11.1	16	88.9	18
Bronchiolitis	1	5.6	17	94.4	18

*The data are reported considering the absolute (n) and relative (%) frequencies of the radiological findings described in the articles included in this study.

Gommans et al. (2014) pointed out that the variability observed among radiological findings of infections caused by NTMs is common and is dependent on the species causing infection. Furthermore, it is suggested that the presence of consolidation on CT images of patients diagnosed with NTM infection is an important predictor of mortality^50^. On the other hand, Hwang et al. (2017) suggested that the presence of fibrocavitary alterations and advanced age are negative prognostic factors for survival of patients with LD by MAC^51^.

These fibrocavitary changes were associated with 39.4% of the cases of LD due to *M. xenopi* based on a study by Hirama et al*.* (2019)^42^. Cowman and Loebinger (2018) had previously identified these fibrocavitary changes in CT scans of patients with NTM infections^40^. In the same study, consolidation was suggested as a common radiological finding^40^. 

 Additionally, studies by Kobashi et al. (2013) and Ueyama et al. (2016) revealed that pneumothorax has been recognized in the lungs of certain patients diagnosed with NTM infections^41^ ^,^ ^52^. Yoon et al. (2016) also suggested that the presence of pneumothorax in CT scans is indicative of the spread of MAC infection but the meaning of this association is not clear^59^. 

The loss of lung mass volume is also a characteristic that is frequently identified among the radiological findings of patients with lung diseases. De Marca et al. (2019) suggested that there is a relationship between mass and lung functions, however, this relationship is not yet fully elucidated^53^.

Although all the findings described above are not specific towards a definitive NTM-LD diagnosis, it is described in the literature that the disease form with nodular infiltrates and bronchiectasis frequently affects the middle lobe and lingula regions^37^ ^,^ ^41^. Representative images of these alterations are shown in Figures 3A and Figure 3B.

## THERAPY

The objective of treatment against NTM-LD is to improve the patient's quality of life by monitoring signs and symptoms. Treatment against NTM infection is long and lasts for 18 to 24 months. The patient is considered cured when he/she is negative for sputum culture for 12 consecutive months^54^. Treatment is a complex decision that involves measuring the benefits and the risk of drug toxicity^11^.

The choice of drug, in general, is directed towards the elimination of the disease-causing NTM species. Microbial susceptibility testing is recommended prior to initiation of treatment. Traditionally, treatment of NTM-LD by MAC consists of the combined use of macrolides (claritormycin or azithromycin), rifampicin and ethambutol^11^. Certain cases of resistance against macrolides and amikacin have already been reported and this resistant or refractory form can be treated alternatively with moxifloxacin, aminoglycosides and clofazimine^11^ ^,^ ^55^.

*M. kansassi* is sensitive to anti-TB drugs and is treated with rifampicin, isoniazid, rifabutin, ethambutol, fluoroquinolones, and amikacin^11^. The pulmonary infection caused by MABSC is progressively slow and its treatment is based on the combined use of intravenous amikacin with cefoxitin or imipenem and an oral macrolide^21^ ^,^ ^29^ ^,^ ^54^. Information on the effectiveness of this treatment is limited and there is evidence that MABSC is resistant to various antibiotics^21^ ^,^ ^54^.

Despite these treatment options, it should be noted that the patient is subjected to side effects, reactions and drug interactions. In the case of macrolides, gastrointestinal discomfort is commonly observed. Use of rifampicin, imipenem and tigecycline might cause hepatotoxicity and rifampicin might alter the metabolism of other medicines such as contraceptives. The use of aminoglycosides, in turn, might cause renal toxicity and hearing impairment^11^. The use of linezoline and ethambutol might cause peripheral neuropathy and ethambutol could also lead to decreased visual acuity^11^. 

In general, it is suggested that treatment should begin within hospital units^11^. In cases where response to chemotherapy is not efficient, surgical resection should be considered and must be conducted by a multidisciplinary technical team^11^ ^,^ ^21^.

## CONCLUSIONS

The increased incidence associated with the complexity of diagnosis makes NTM-LD a public health concern. Correct and rapid diagnosis is critical for the proper and effective choice of treatment. Currently, diagnostic criteria include clinical evidence, microbiological identification, and radiological findings. NTM-LD symptoms are similar to other pulmonary infections. Microbiological identification is not always possible. Based on the radiological alterations observed in NTM-LD, bronchiectasis, cavitations and pulmonary nodules are predominantly found. Although these alterations overlap with other lung diseases, when they are predominantly found in the middle lobe and lingula, mycobacteriosis should be suspected.
